# A spatio-temporal analysis of fire occurrence patterns in the Brazilian Amazon

**DOI:** 10.1038/s41598-023-39875-z

**Published:** 2023-08-05

**Authors:** Fernanda Valente, Márcio Laurini

**Affiliations:** Department of Economics, University of São Paulo, Ribeirão Preto, São Paulo 100190 Brazil

**Keywords:** Environmental sciences, Natural hazards, Mathematics and computing

## Abstract

Wildfires in the Amazon significantly impact the forest structure and carbon cycle. Understanding the patterns of fire occurrence is crucial for effective management. A novel spatio-temporal point process framework was used to analyze changes in fire occurrence patterns in the Brazilian Amazon. A dynamic representation of a Log Gaussian Cox process was used to model the intensity function, which was decomposed into trend, seasonality, cycles, covariates, and spatial effects. The results show a marked decrease in long-term fire occurrence movements between the start of the sample and 2012, followed by an increase until the end of the sample, attributed to governance measures and market mechanisms. Spatial variability of fire occurrence rates in the Brazilian Amazon was successfully captured, with regions having more dry seasons experiencing higher fire occurrence rates. This analysis provides valuable insights into fire occurrence patterns in the Amazon region and the factors driving them.

## Introduction

The Amazon biome is widely recognized as a significant global asset due to its extraordinary biodiversity, encompassing more than half of the world’s rainforests and supporting a quarter of all terrestrial species^1^. Moreover, the Amazon rainforest plays a vital role in mitigating global warming by serving as a carbon reservoir through carbon storage in biomass and soils^2^. Furthermore, the evaporation and precipitation processes in the Amazonia region have far-reaching implications for the global atmospheric circulation, influencing climate patterns not only in South America but also across the Northern Hemisphere^3,4^. While the Amazon rainforest spans multiple countries, approximately 60% of the Amazon Basin falls within Brazil, with the administrative region known as Legal Amazon covering nine Brazilian states, accounting for 61% of the Brazil national territory.

Fire occurrences, stemming from both natural and human activities, are significant disturbances in the Amazon region, impacting atmospheric composition^5,6^, forest structure and composition^7^, and the carbon cycle. Generally, fire incidence in the Amazon rainforest is influenced by land use, land cover, and climate patterns. Notably, the majority of changes in land cover and human activities are concentrated in the southern and eastern areas of the Brazilian Amazon region, often referred to as the “arc of deforestation” by^8^. The expansion of roads and agriculture in the Legal Amazon began in the early 1970s with the construction of the Transamazon Highway, resulting in high rates of deforestation. For instance, between 1980 and 1990, deforestation rates in the Legal Amazon substantially increased, leading to the clearance of approximately 225,000 km$^{2}$ of forest. Concurrently, the length of paved roads increased by over 100%, while unpaved roads saw a growth of approximately 460%^9^.

From an economic perspective, fire incidents in the Amazon region incur various costs, with both private and social consequences. In rural areas, the primary losses occur when fires get out of control, spreading to pastures and forested areas. Additionally, fire-related losses can have wider social impacts, including the release of carbon into the atmosphere, which affects global climate patterns and leads to adverse health outcomes. These consequences impose direct and indirect costs on society, such as medical expenses, loss of labor, and reduced utility^10,11^.

There are various factors that can influence the occurrence patterns of fires in the Amazon region. Previous studies have indicated the impact of dry conditions on the risk of forest fires^12,13^, as well as the effects of deforestation and fragmentation on the regional climate. These effects include a significant increase in mean surface temperature and a decrease in annual evapotranspiration and precipitation, which can further elevate the risk of fires^14,15^. Agricultural expansion has also been identified as a contributing factor to changes in forest fire patterns^16^, as fires are commonly used by Brazilian farmers as a cost-effective method to expand agricultural frontiers and maintain and rejuvenate pastures. Additionally, climate change, including shifts in circulation patterns and increased anomalies like El Niño events, exacerbates the occurrence of extreme dry seasons in the Amazonia region^17–19^, which alters vegetation structure and potentially transforms the forest from being highly resistant to fire ignition to extensively flammable^20^, ultimately leading to an increase in future burning frequency. Moreover, there is evidence to suggest that forest fires create a positive feedback loop in terms of fire susceptibility, fuel loading, and fire intensity, making recurrent fires more likely and severe^21,22^.

One approach to assess changes in climate-related events, such as fire occurrence, is to estimate permanent and periodic components^23–25^, using statistical tools to decompose the observed temporal variability into trend, seasonal, and cyclical components. However, existing methods used for extracting trends, seasonality, and cyclical components face certain challenges when it comes to performing inference procedures on climate-related issues. Firstly, these models are not well adapted to the dimensionality of the data sources commonly used in climatology^26^, and they do not adequately account for the spatial heterogeneity of climate effects^25^. An alternative approach to address these issues is to combine elements of structural time series decomposition with spatio-temporal models that incorporate continuous spatial random effects. This approach can be seen as a process of decomposing geostatistical time series into a combination of trend, seasonal, and cyclical components, as well as the effects of additional covariates^25,27,28^.

The objective of this study is to analyze changes in the occurrence patterns of fires in the Legal Amazon and the Amazon biome within the framework of spatio-temporal point processes. To achieve this, we propose a methodology that extends the trend-cycle decomposition in spatio-temporal models to spatio-temporal point pattern data. Specifically, we suggest using a dynamic representation of a Log Gaussian Cox process (LGCP), where the intensity function is modeled by decomposing the components into trends, seasonality, cycles, covariates, and spatial effects^25,27,28^. This formulation is valuable for identifying potential changes in the occurrence intensity over time, such as permanent changes in fire occurrence, and capturing seasonal and cyclical effects.

The Log Gaussian Cox process (LGCP) is a specific instance of the Cox process, where the log-intensity function follows a Gaussian random field. However, due to the stochastic nature of the LGCP, fitting this model can be computationally demanding^29^. To address this challenge and achieve efficient estimation, we employ the stochastic partial differential equation (SPDE) approach^30^ to transform the initial Gaussian random field (GRF) into a Gaussian Markov Random Field (GMRF), which is characterized by sparse matrices. Additionally, the resulting Bayesian hierarchical model is compatible with the integrated nested Laplace approximations (INLA) framework^31^, which further enhances computational efficiency.

In this study, we present the analysis results of fire occurrence data in the Legal Amazon and the Amazon biome, spanning from July 2002 to December 2022. Our database comprises daily fire reports obtained from the Moderate-Resolution Imaging Spectroradiometer (MODIS), including information such as spatial coordinates and temporal instances of fire events. Furthermore, we incorporate explanatory variables to account for the main fixed effects related to climatic conditions and soil usage. Our findings demonstrate a distinct long-term trend in fire occurrence, with a notable decline from the beginning of the dataset until 2012, followed by a subsequent increase that persists until the end of the observation period. These patterns could be attributed to governance interventions and market mechanisms. Moreover, our model effectively captures the spatial variations within the Legal Amazon, particularly in regions classified as wet tropical (Am), characterized by a dry season occurring between August and November (third and fourth quarters), as well as tropical regions with a dry season (Aw). Conversely, in the western Amazon, where the climate is predominantly tropical without a dry season (Af), the variability is relatively low.

The structure of our paper is as follows: in “Methodology and data”, we introduce the proposed methodology and describe the data used. “Results presents the obtained results, while “Discussion” discusses and interprets these findings. Finally, we provide our concluding remarks in “Conclusion”, and additional results are presented in the supplement of the article.

## Methodology and data

### Methodology

Among models for the spatial point process, the Poisson process is considered the most fundamental structure^32^. However, its application is limited due to its simplistic nature, even when assuming a non-homogeneous distribution in space through a function of deterministic intensity^32^. The limitations are associated with the absence of possible sources of uncertainty and the conditional independence property of the Poisson process. A related, yet more flexible structure is the Log Gaussian Cox process (LGCP), which is a hierarchical model where the process is assumed to be Poisson conditioned on the intensity function at the first level, and the log of the intensity function is assumed to follow a Gaussian field at the second level^32^.

Fitting the LGCP model poses a computational challenge due to its doubly-stochastic property. In the Bayesian framework, the conditional autoregressive approach provides an alternative for performing inference procedures and can be fitted using the Integrated Nested Laplace Approximation (INLA)^33^. However, this approach is based on regular lattices over the observation window^29^, which may be highly inefficient as it requires constructing a fine grid. For spatial models that combine a Gaussian random field (GRF) with a Matérn correlation structure, the stochastic partial differential equations (SPDE)^30,34^ approach offers a solution to the inefficiency problem in estimation under the INLA method. The key idea is to leverage the fact that a GRF with Matérn covariance function is a solution to a SPDE, and the SPDE representation, combined with a basis representation, is used to construct a discrete approximation of the continuous field over the vertices of a two-dimensional mesh covering the spatial domain^29^. In other words, the SPDE approach aims to approximate the initial Gaussian field as a Gaussian Markov random field, providing the advantage of computationally efficient methods due to the sparse matrix representation of GMRFs.

In this paper, we use a spatio-temporal representation of spatial point processes with stochastic intensity by decomposing the intensity function into components that vary both in time and space. Specifically, we adopt an LGCP structure where the intensity function is decomposed into trend, seasonal, and cyclical components, along with spatial random effects^25^. This decomposition enables us to identify permanent changes, as well as cyclical and seasonal effects. To perform inference procedures, we employ the SPDE approach, allowing us to utilize Bayesian inference methods based on INLA. Our implementation follows the general structure proposed in Valente et al.^28^, which uses a basic version of the model without the inclusion of covariates to analyze spatio-temporal patterns of fires in Australia, and thus our formulation can be interpreted as a generalization of^28^. We provide a brief description of the SPDE approach, and further details can be found in Lindgren et al.^30^ and Simpson et al.^29^.

Spatio-temporal data can be represented as realizations of a stochastic process (a random field) indexed by both space and time dimensions.

1 $$\begin{aligned} Y(s,t)=\{y(s,t) \mid (s,t) \in D \times T \in {\mathbb {R}}^2 \times {\mathbb {R}}\} \end{aligned}$$

where $D$ is a subset of ${\mathbb {R}}^2$, $T$ is a subset of ${\mathbb {R}}$, $s$ encodes a spatial coordinate and $t$ denotes a time index. With this formulations, we represent a spatio-temporal LGCP as

2 $$\begin{aligned} Y(s,t)&=Poisson(e(s,t) exp(\lambda (s,t)), \nonumber \\ \lambda (s,t)&= z(s,t)\beta + \xi (s,t) \nonumber \\ \xi (s,t)&= \Phi \xi (s,t-1)+\omega (s,t) \end{aligned}$$

where *Y*(*s*, *t*) is the counting of fire occurrences in a location *s* and in time *t*, *e*(*s*, *t*) is the exposure offset for the region *s*, $z(s,t)$ is a set of observed covariates in the location $s$ and time period $t$, and $\xi (s,t)$ are the spatial random effects, which follow a spatially continuous Gaussian process $\omega (s,t)$ given by

3 $$\begin{aligned} Cov(\omega (s,t) \omega _(s',t'))= {\left\{ \begin{array}{ll} 0 \quad \text {if } t \ne t' \\ \sigma ^2 C(h) \quad \text {if } t=t' \end{array}\right. } \quad \text {for } s\ne s' \end{aligned}$$

where $C(h)$ is a covariance function of the Matérn class

4 $$\begin{aligned} C(h) = \frac{2^{1-\nu }}{\Gamma (\nu )}(\kappa h)^{\nu }K_{\nu }(\kappa h) \end{aligned}$$

where $h=s-s'$ is the Euclidean distance between locations $s$ and $s'$, $\kappa >0$ is a spatial scale parameter, $\nu >0$ is parameter controlling the smoothness of the process and $K_{\nu }$ is the modified Bessel function. The marginal variance $\sigma ^2$ is obtained as:

5 $$\begin{aligned} \sigma ^2 = \frac{\Gamma (\nu )}{4\pi \kappa ^{2\nu }\tau ^2\Gamma (\nu + \frac{d}{2})} \end{aligned}$$

with $\tau$ being a scale parameter and $d$ is the spatial dimension. Additionally, we use a reparameterization in terms of $\log \tau$ and $\log \kappa$ for the covariance function^30^:

6 $$\begin{aligned} \log \tau&= \frac{1}{2}\log \left( \frac{\Gamma (\nu )}{\Gamma (\alpha )(4\pi )^{d/2}}\right) -\log \sigma -\nu \log \rho \nonumber \\ \log \kappa&=\frac{\log (8\nu )}{2}-\log \rho \end{aligned}$$

and $\rho = \frac{(8\nu )^{1/2}}{\kappa }$. This representation is advantageous since, given $\nu$, it is necessary to estimate only two parameters.

Assuming a bounded region $\Omega \in {\mathbb {R}}^2$, it follows^29^ that the likelihood for an LGCP associated with data $Y = \{s_i \in \Omega : i = 1, \ldots , n; t=1,\ldots ,T\}$ is

7 $$\begin{aligned} \pi (Y \mid \lambda ) = \exp \left( \mid \Omega \mid - \int _{\Omega } \lambda (s,t)ds\right) \prod _{t=1}^T\prod _{i=1}^{n_t}\lambda (s_i,t). \end{aligned}$$

Due to the doubly-stochastic property of the intensity function, the likelihood in (7) is analytically intractable, as discussed by^29^. Since the term $\omega (s,t)$ corresponds to a GF with Matérn covariance, Simpson et al.^29^ shows that it is possible to adopt SPDE formulation to approximate the GF with GMRF. Using the fact that a GF x(s) with the Matérn covariance function is equivalent to the stationary solution to the linear fractional SPDE^30,34^:

8 $$\begin{aligned} (\kappa - \Delta )^{\alpha /2}x(s)=W(s), \quad s \in {\mathbb {R}}^d, \quad \alpha = \nu +d/2, \quad \kappa>0, \quad \nu >0 \end{aligned}$$

where $\Delta = \sum _{i=1}^d \frac{\partial ^2}{\partial s^2_i}$ is the Laplacian operator and $W(s)$ is a spatial white noise. Therefore, to find a GMRF approximation of a GF, it is necessary to find the stochastic weak solution of a SPDE, which can be constructed through finite method elements (FEM)^30^. Using this property, the approximated SPDE solution is

9 $$\begin{aligned} \omega (s,t) \approx \tilde{\omega }(s,t) = \sum _{j=1}^n w_j \varphi _j(s,t) \end{aligned}$$

where $n$ is the number of vertices of the triangulation, $\{w_j\}^n_{j=1}$ are the weights with Gaussian distribution and $\{\varphi _j\}^n_{j=1}$ are the basis functions defined for each node on the mesh, following the definitions used by^30^. To summarize, the concept involves computing the weights $\{w_j\}$ that define the field values at the vertices, while the values within the triangles are determined through linear interpolation^30^. In this approach, the basis functions are selected as piecewise linear functions within each triangle.

10 $$\begin{aligned} \varphi _l (s,t)= {\left\{ \begin{array}{ll} 1 \quad \text {at vertex } l \\ 0 \quad \text {elsewhere} \end{array}\right. } \end{aligned}$$

The stochastic weak solution of (8) is found by imposing

11 $$\begin{aligned} \{\langle \phi , (\kappa ^2-\Delta )^{\alpha /2}\omega \rangle \}_{\Omega }\overset{d}{=}\{\langle \phi , W\rangle \}_{\Omega }, \end{aligned}$$

where $\{\phi _i(s), i=1,\ldots ,m\}$ are test functions and “$\overset{d}{=}$” denotes equality in distribution. Replacing (9) in (11) gives us

12 $$\begin{aligned} \{\langle \phi _i, (\kappa ^2-\Delta )^{\alpha /2}\varphi _j\rangle \}_{\Omega } \textbf{w}\overset{d}{=}\{\langle \phi _i, W\rangle \}_{\Omega }, \end{aligned}$$

for $i = 1,\ldots ,m$, and $m$ is the number of test functions. The finite dimensional solution is the distribution for the Gaussian weights in Eq. (9) that fulfils (12) for a certain set of test functions, with $m=n$. When $\phi _k = (\kappa ^2=\Delta )^{1/2}\varphi _k$ for $\alpha =1$ and $\phi _k = \varphi _k$ for $\alpha =2$, these two approximations are denoted as *least squares* and *Galerkin* solutions, respectively. Assuming $\alpha =2$ and $\phi _k=\varphi _k$ yields

13 $$\begin{aligned} \left( \kappa ^2 \{\langle \varphi _i, \varphi _j \rangle \}+\{\langle \varphi _i, -\Delta \varphi _j\rangle \}\right) \textbf{w} \overset{d}{=}\ \{ \langle \varphi _i, W \rangle \}. \end{aligned}$$

Define the $n \times n$ matrices, **C** and **G** as

14 $$\begin{aligned} C_{ij}&= \langle \varphi _i, \varphi _j \rangle \nonumber \\ G_{ij}&= \langle \nabla \varphi _i, \nabla \varphi _j \rangle , \end{aligned}$$

then a weak solution to (8) is given by (9), where

15 $$\begin{aligned} (\kappa ^2\textbf{C}+ \textbf{G})\textbf{w} \sim N(0,\textbf{C}) \end{aligned}$$

and the precision of the weights, **w**, is

16 $$\begin{aligned} \textbf{Q}_{\alpha =\textbf{2}}= (\kappa ^2\textbf{C}+ \textbf{G})^T\mathbf {C^{-1}}(\kappa ^2\textbf{C}+ \textbf{G}). \end{aligned}$$

Although $G_{ij}$ and $C_{ij}$ are sparse matrices, $\textbf{C}^{-1}$ is dense. The solution is to replace $C_{ij}=\langle \varphi _i, \varphi _j \rangle$ by the diagonal matrix $C_{ii}=\langle \varphi _i, 1 \rangle$, that yields a Markov approximation. Therefore, **w** is a Gaussian Markov Random Field with precision (16).

By replacing the GF $\omega (s,t)$ with the GMRF approximation $\tilde{\omega }(s,t)$ in Eq. (2), and approximating the integral in Eq. (7) using a quadrature rule, the resulting approximate likelihood consists of $(n + n_t)T$ independent Poisson random variables, where $n$ is the number of vertices and $n_t$is the number of observed point processes^29^. By obtaining the LGCP likelihood approximation, it is possible to perform inference procedures through the INLA algorithm, which provides accurate and efficient approximations for Bayesian hierarchical models that can be represented as latent Gaussian models. For details about the INLA method, refer to^31^.

The dynamic formulation proposed in this paper is a generalization of the formulation given in Eq. (2). In this case, we include the components $\mu _t$ and $s_t$ as follows:

17 $$\begin{aligned} Y(s,t)&=Poisson(e(s,t) exp(\lambda (s,t)), \nonumber \\ \lambda (s,t)&= \mu _t + s_t + z(s,t)\beta + \xi (s,t) \nonumber \\ \Delta ^2 \mu _t&= \mu _t-2 \mu _{t+1}+\mu _{t+2} \nonumber \\ s_t&= s_{t-4} + \eta _s \nonumber \\ c_t&= \theta _1c_{t-1}+ \theta _2c_{t-2}+ \eta _c \nonumber \\ \xi (s,t)&= \Phi \xi (s,t-1)+\omega (s,t) \end{aligned}$$

where $\mu _t$ is the long term (permanent) trend modeled as a second-order random walk (RW2), also known as the local-trend model. The $s_t$ represents the seasonal effects, which is based on a seasonal autoregressive model. The $c_t$ is a cycle component represented by a second-order autoregressive process with possible complex roots, which allows to capture cyclic patterns if the roots are in the complex region of the plane^25^. The $\eta _{\mu }$, $\eta _c$ and $\eta _s$ are independent innovations with $\eta _{\mu }\sim N(0, \sigma ^2_{\eta _{\mu }})$, $\eta _{c}\sim N(0, \sigma ^2_{\eta _{c}})$ and $\eta _s\sim N(0, \sigma ^2_{\eta _{s}})$. In all estimation procedures, we use default priors for the SPDE model in the R-INLA package implementation, which is available upon request from the authors.

### Data

In this paper, we use daily data of fire occurrence in the Legal Amazon and Amazon biome from MODIS Thermal Anomalies/Fires between July 2002 and December 2022, which provides information such as fire occurrences (day/night), fire location, the logical criteria for the fire selection, and detection confidence. The Brazilian Legal Amazon and the Brazilian Amazon Biome are related but distinct concepts. The Brazilian Legal Amazon is a region defined by Brazilian law, comprising nine states: Acre, Amapá, Amazonas, Pará, Rondônia, Roraima, Mato Grosso, Tocantins, and parts of Maranhão. This region covers approximately 59% of Brazil’s total land area and is characterized by a large forested area, including the Amazon rainforest. On the other hand, the Brazilian Amazon Biome is a large tropical forest that covers most of the Amazon Basin in South America. The Brazilian Amazon Biome includes not only the Brazilian Legal Amazon but also other regions in Brazil that are part of the biome. The Brazilian Legal Amazon is a political-administrative region defined by Brazilian law, while the Brazilian Amazon Biome is an ecological and biogeographic region defined by its natural characteristics.

In order to provide better interpretations of the results, we use a quarterly aggregation of the daily data. In addition, from the computational aspect, the use of a very high frequency could lead to numerical problems in the estimation and inference processes since the dimension of the spatio-temporal covariance matrix is given by the Kronecker product between the time and spatial dimensions. We present here the results for the Legal Amazon, and the results for the Amazon Biome are presented in the next section.

**Figure 1:**
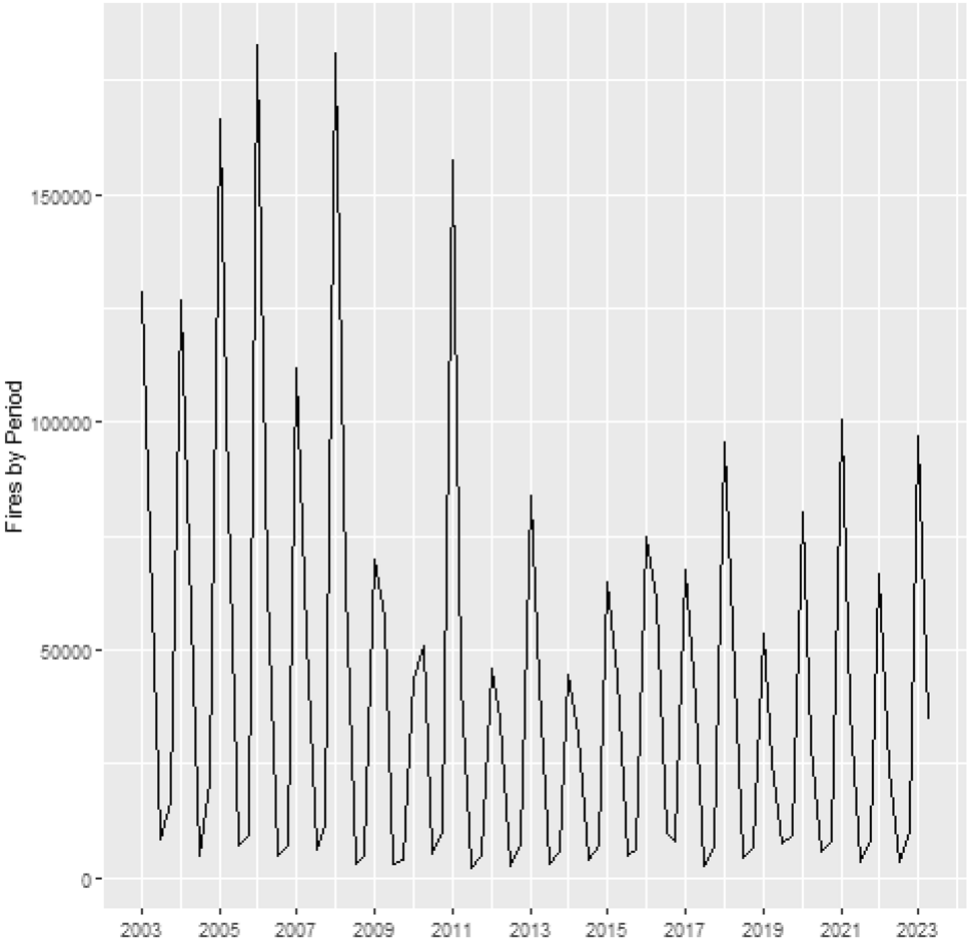
Fires in Legal Amazon by quarter between 2002 and 2022.

**Figure 2:**
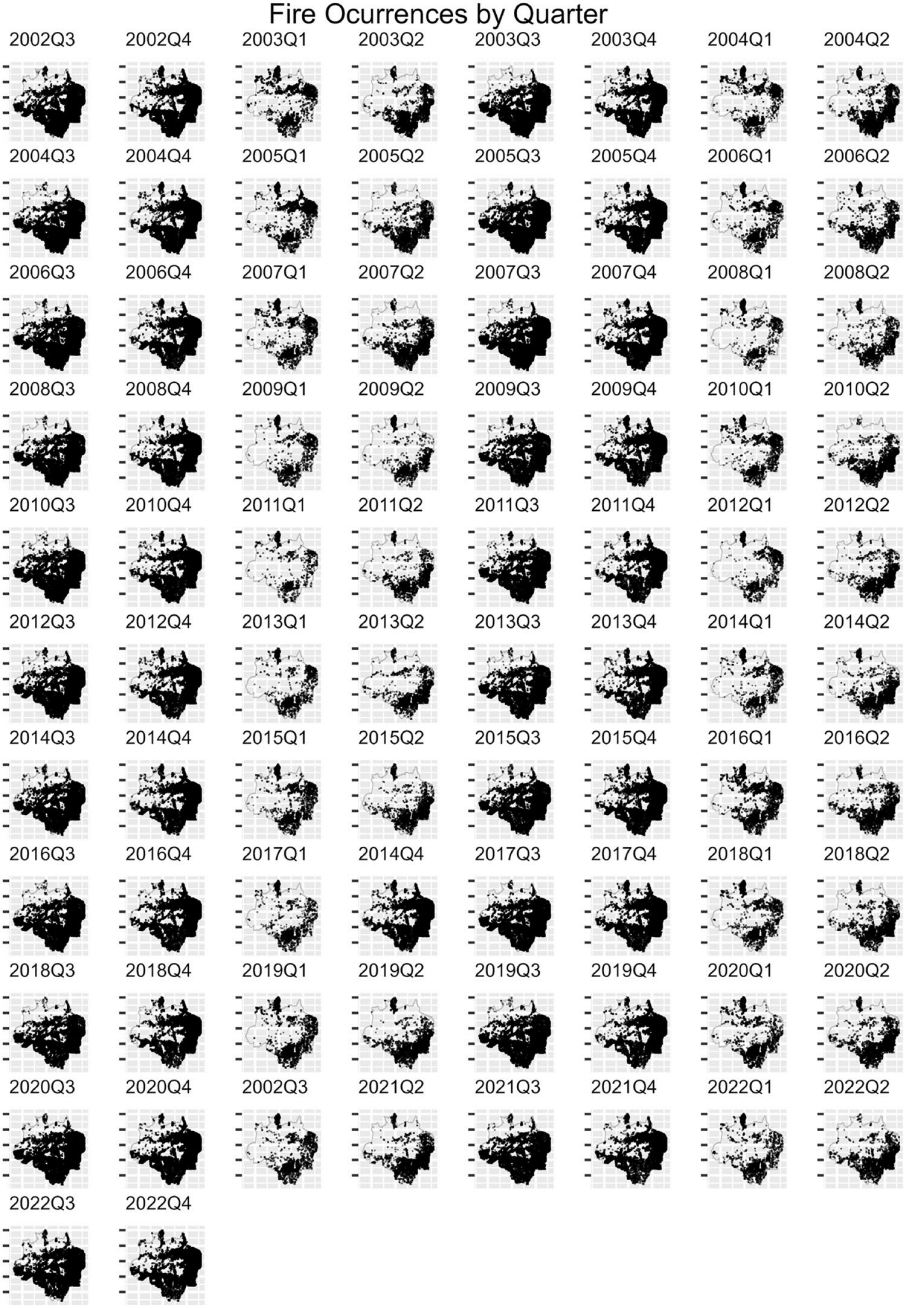
Spatial distribution of fires in Legal Amazon between 2002 and 2022.

To illustrate, Fig. 1 provides the number of fire events over time in the Legal Amazon, while Fig. 2 shows a graphical distribution of the fires over time and space. From July to October 2005 large areas of the Amazon region experienced one of the strongest drought of the past 100 years^19^. The event in 2005 was driven by elevated tropical North Atlantic sea surface temperatures associated with a weaker cold anomaly in the South Atlantic^19,35^, and caused intense forest fire. After the peak in 2005, the fire occurrence in the Legal Amazon decreased until 2012, whereas from 2013 to 2022 forest fires increased (see Fig. 1). The spatial distribution of fire occurrence shows that forest fires are more concentrated in the region called “arc of deforestation”, an area that extends from Maranhão to Acre, but with a pattern of increasing toward central areas. Additionally, it is possible to note that most of the fire events occur during the third and fourth quarter, the dry season (May–October).

Since our data base includes fire occurrence of different causes, such as human sources (deliberately or accidentally), and natural causes, it is important to include explanatory variables in the analysis to control the main fixed effects related to climatic conditions and to control for possible use of the soil in agricultural and livestock activities. Thus, to reach our goal, we include explanatory variables, as the Köppen climate Classification, which classifies the climate in a certain region by types (see Table 1 in Supplementary Material) that are characterized by two or three characters, where the first indicate the climate zone defined by the temperature and rainfall, the second is defined by the rainfall distribution, and the third considers the sea seasonal temperature variation^36^. According to Köppen Classification, the climate in the Legal Amazon is mostly wet tropical (Am) in the central areas, tropical with dry winter (Aw) in the Southeastern Amazon and tropical without dry season (Af) in Western Amazon.

We also include MapBiomas Collection 7 for Amazon that contains annual land use and land cover maps (LULC), that classifies the Amazon Biome into 7 different classes of land cover/land use including forest formation, savanna formation, wetland, grassland formation, pasture, agriculture, other non-vegetated area, non-observed, and water bodies (Available at https://mapbiomas.org/).

Evidence of intentional fire can be seen through the proximity of fire outbreaks and highways, as proximity to highways implies human accessibility and lower transportation costs for agricultural and livestock production. Therefore, as explanatory variable, we also include the distance of fire occurrence from federal and state highways. The data base containing the location of federal and state highways is provided by *Departamento Nacional de Infraestrutura de Transportes* (DNIT) and *Empresa de Planejamento e Logística* (EPL).

Lastly, we include rainfall and maximum temperature information which were constructed using the time series of the monitoring stations provided by *Agência Nacional de Águas* and *Instituto Nacional de Meteorologia* (INMET), whereas the maximum temperature data were obtained based on the information provided by the INMET. In both cases, we used the spatially continuous projections for each period in the sample, which were calculated based on the methodology proposed by^37^.

## Results

In this section, we report the results obtained with the estimation of the model described in “ Methodology and data” to map changes in spatial and temporal patterns of fire occurrence in the Brazilian Amazon. We perform inference procedures based on the specification described in Eq. (2). Thus, the estimated parameters are the precision of the trend component ($1/\eta _{\mu }$), seasonal component ($1/\eta _{s}$), and cycle component ($1/\eta _{c}$), the parameters of the second-order autoregressive process of the cycle (PACF1 and PACF2), the parameters associated with the set of observed covariates ($\beta$), the parameters of spatial covariance ($\log \tau$ and $\log \kappa$), and the parameter of spatial time dependence ($\Phi$).

Table 1 reports the estimated parameters. As might be expected, the results indicate a negative relation between the distance to roads and the fire occurrence. The importance of the highways as a prime driver of fire occurrence and deforestation at local scales has been discussed in the literature, showing that the roads play important roles facilitating transformation of land-use practices, creating fresh access to new settlements in frontier regions, and reducing transportation costs in earlier settled areas^9,38^.

Regarding the rainfall and temperature covariates, the results indicate a negative relationship between rainfall and the intensity of fire occurrences and, on the other hand, higher temperatures are related with higher incidence of fires, according to the results.

**Table 1:** Estimated parameters for the Legal Amazon.

	Mean	SD	0.025quant	0.5quant	0.975quant	Mode
Fixed effects						
Distance highways	− 0.012	0.001	− 0.014	− 0.012	− 0.010	− 0.012
Temperature	0.155	0.011	0.132	0.155	0.177	0.155
Rainfall	− 0.006	0.001	− 0.007	− 0.006	− 0.005	− 0.006
Köppen 1 (Cwa)	0.012	0.248	− 0.474	0.012	0.497	0.012
Köppen 2 (Am)	0.252	0.061	0.134	0.252	0.371	0.252
Köppen 3 (Af)	0.219	0.094	0.035	0.219	0.404	0.219
Köppen 4 (Cfa)	0.153	0.254	− 0.344	0.153	0.650	0.153
Köppen 10 (As)	0.265	0.159	− 0.045	0.265	0.576	0.265
Köppen 12 (Aw)	0.444	0.143	0.165	0.444	0.724	0.444
Forest formation	− 0.024	0.055	− 0.131	− 0.024	0.084	− 0.024
Savanna formation	0.056	0.084	− 0.109	0.056	0.220	0.056
Mangrove	0.133	0.152	− 0.166	0.133	0.432	0.133
Wetland	0.205	0.112	− 0.015	0.205	0.426	0.205
Grassland	0.151	0.078	− 0.003	0.151	0.304	0.151
Pasture	0.076	0.079	− 0.078	0.076	0.230	0.076
Mosaic of uses	0.078	0.128	− 0.173	0.078	0.328	0.078
Beach, dune and sand spot	0.542	0.248	0.055	0.542	1.029	0.542
Other non vegetated areas	− 0.541	0.177	− 0.888	− 0.541	− 0.195	− 0.541
River, lake and ocean	0.154	0.066	0.024	0.154	0.284	0.154
Soybean	0.088	0.177	− 0.258	0.088	0.435	0.088
Other temporary crops	0.116	0.169	− 0.214	0.116	0.447	0.116
Random effects						
Precision for trend	5.270	0.185	4.897	5.271	5.633	5.284
Precision for seasonality	1.059	0.082	0.933	1.049	1.251	1.010
PACF4 for seasonality	0.136	0.039	0.074	0.132	0.224	0.112
Precision for cycle	4.673	0.161	4.344	4.676	4.983	4.694
PACF1 for cycle	0.308	0.020	0.264	0.309	0.344	0.314
PACF2 for cycle	− 0.366	0.017	− 0.403	− 0.365	− 0.336	− 0.360
Log $\tau$	− 2.135	0.007	− 2.148	− 2.136	− 2.120	− 2.137
Log $\kappa$	− 0.035	0.008	− 0.052	− 0.034	− 0.022	− 0.031
Group $\Phi$	0.842	0.003	0.837	0.842	0.847	0.841

Based on the Köppen classification, the climate of the Legal Amazon is predominantly characterized by a wet climate (Af), experiencing precipitation throughout the year. It also exhibits a monsoon climate (Am) with an annual total precipitation exceeding 1500 mm, and a dry season occurring from August to November. Additionally, the region features a tropical climate with a distinct dry season (Aw). As expected, obtained results suggest that the types of climates with dry season (Am, As, and Aw) have higher influence on fire occurrence than those without dry season (Cwa, Af, and Cfa). Our analysis associated to land cover classifications shows a positive relation between fire occurrence and savanna formation, mangrove, wetland, grassland, pasture, mosaic of uses, sand spot, water bodies, soybean, and other temporary crops. On the other hand, the estimated parameters indicate a negative relation between fire occurrence and forest formation and other non vegetated areas.

Regarding the random effects, the precision parameters represent the variability associated with the trend, seasonal and cycle components, where high values indicate low variability. Based on the results reported in Table 1, it is possible to note a high precision associated with the cycle component as well as the trend component, whereas the seasonality component shows a relatively minor precision.

**Figure 3:**
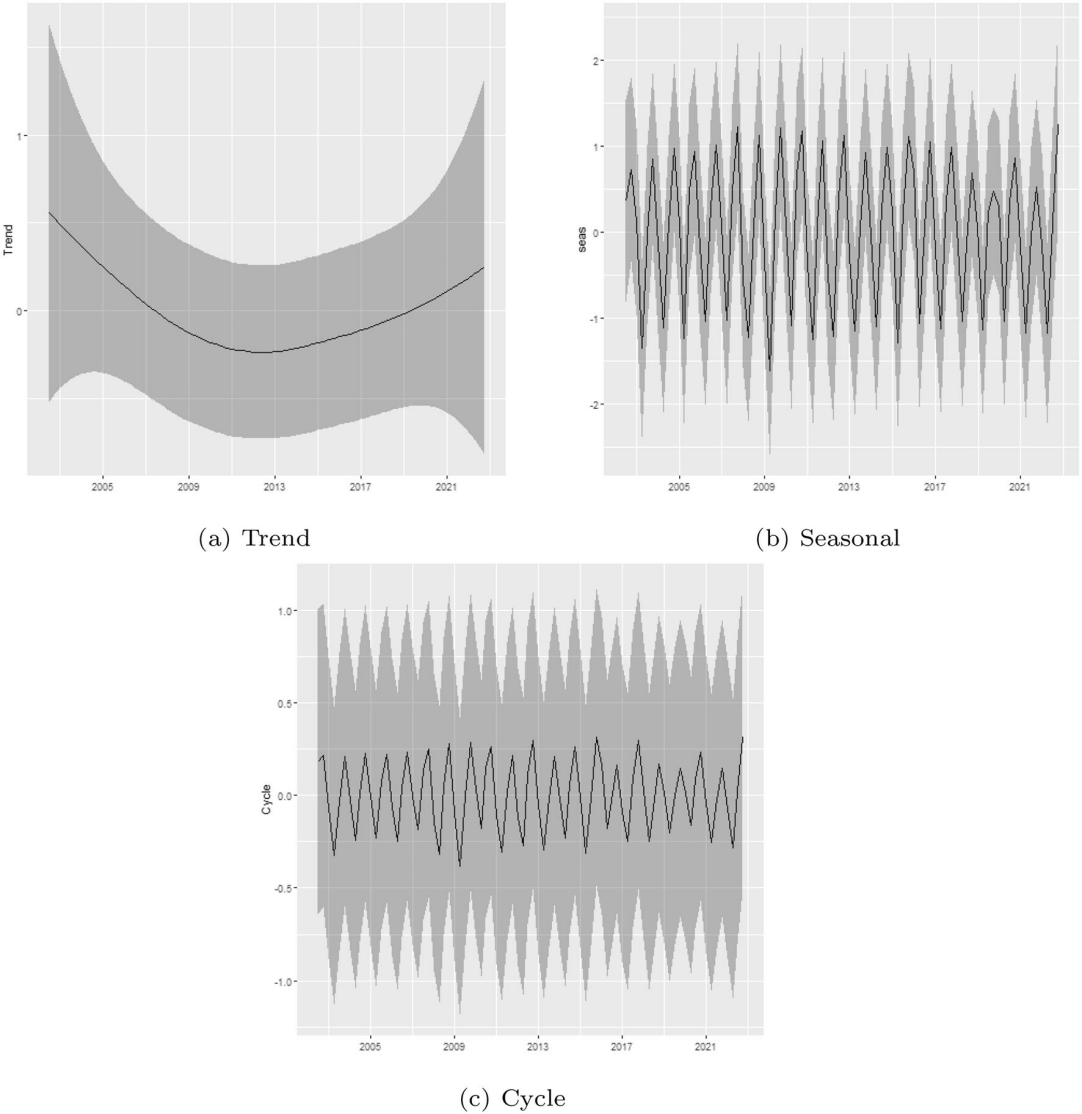
Trend, seasonal and cycle decomposition of fire occurrences in the Legal Amazon.

A primary empirical motivation for the present study was to assess the existence of changes in the patterns of fire occurrence in the Legal Amazon. To better understand the results, we plotted the estimated trend, seasonal and cycle components (posterior mean and 95% Bayesian credibility interval; see Fig. 3). The trend component exhibit a marked decrease between the beginning of the sample and 2012, followed by an increase that extends to the end of the sample. Regarding the cycle and seasonal components, based on Fig. 3, it is possible to note that both are quite stable, and the model does not indicate relevant changes in those components.

**Figure 4:**
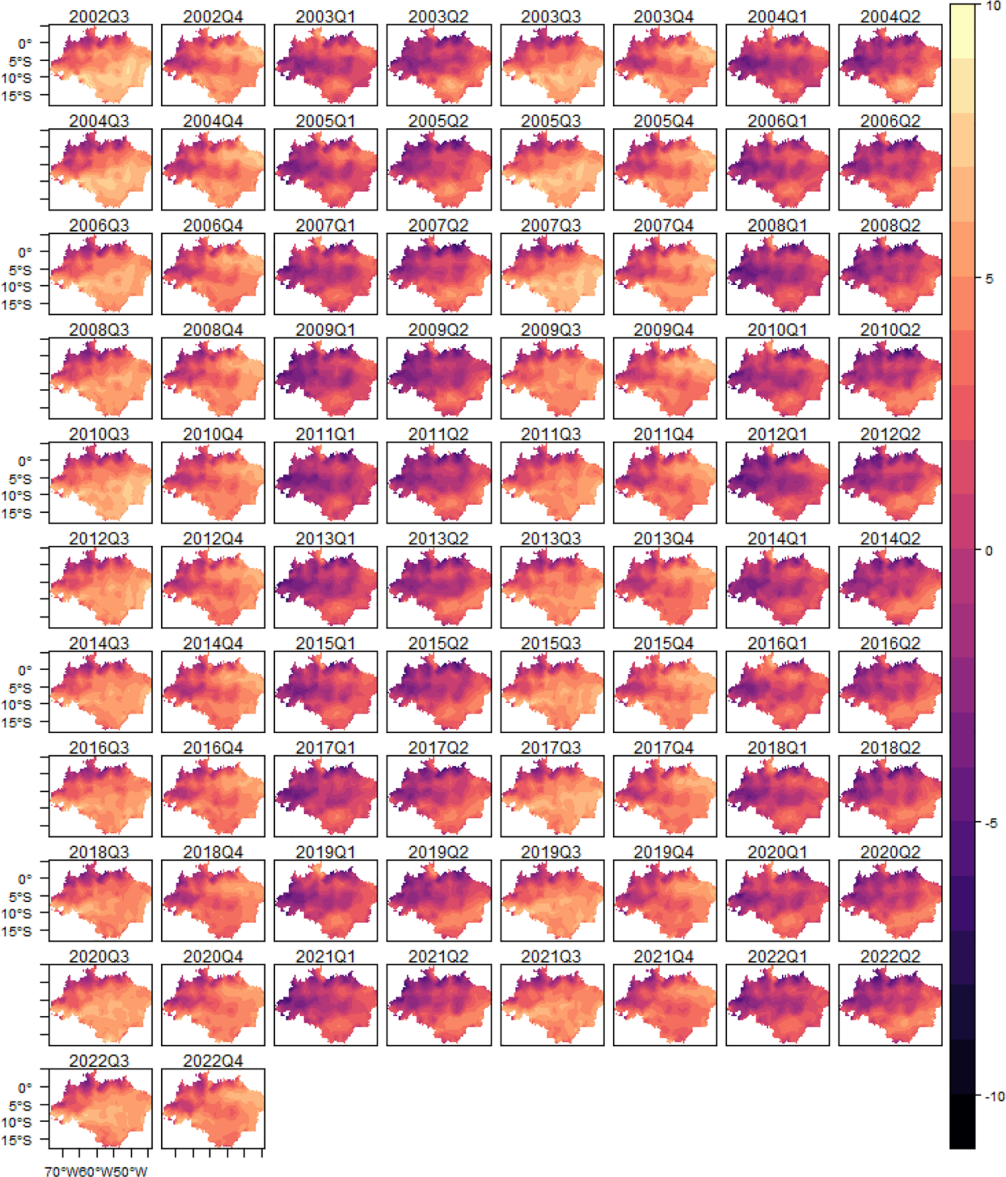
Spatial random effects-Legal Amazon.

The spatial heterogeneity of the fire occurrence in the Legal Amazon can be better seen through the estimated spatial random effect (posterior mean of estimated spatial random effect; see Fig. 4).

**Figure 5:**
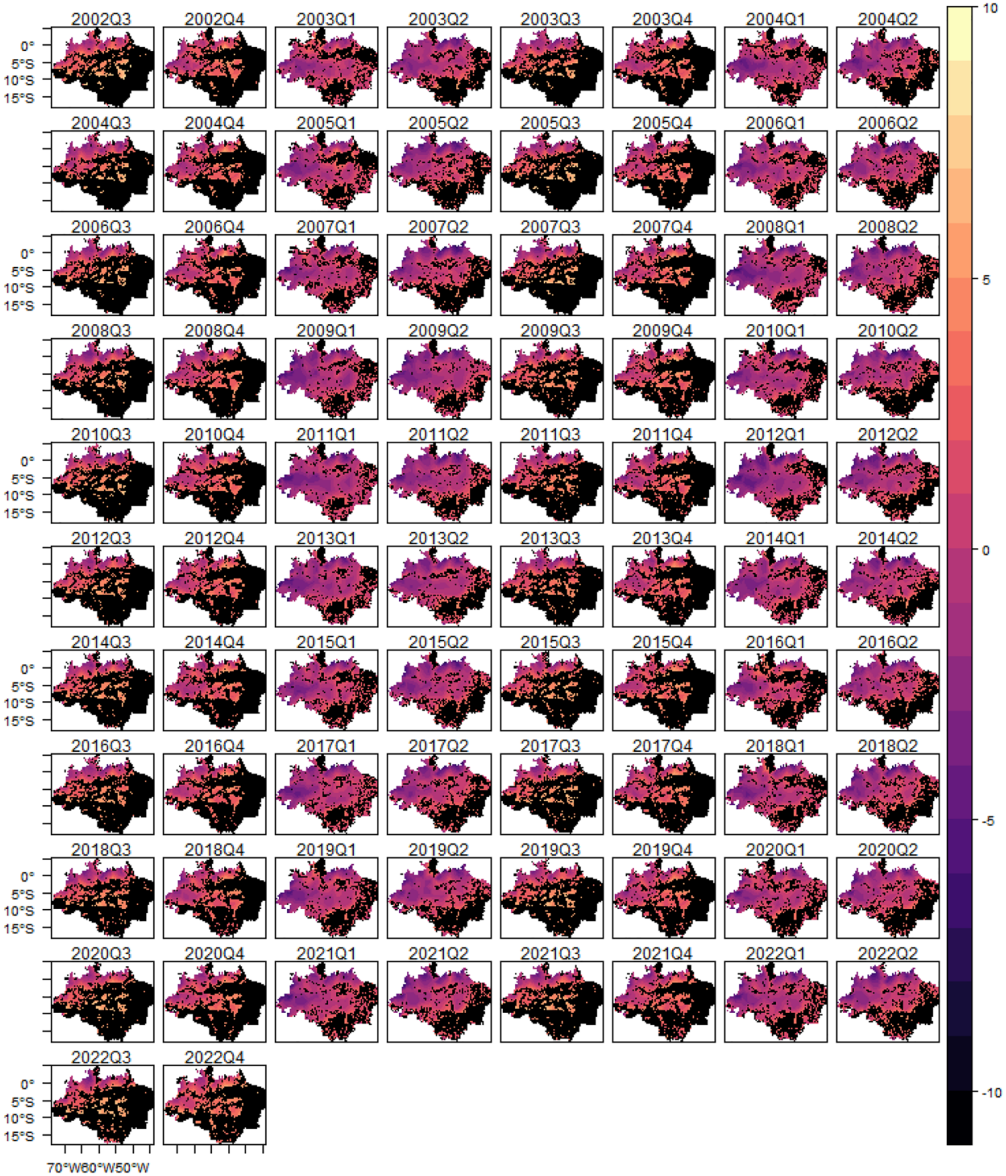
Estimated log-intensity function and observed fire occurrence-Legal Amazon.

**Figure 6:**
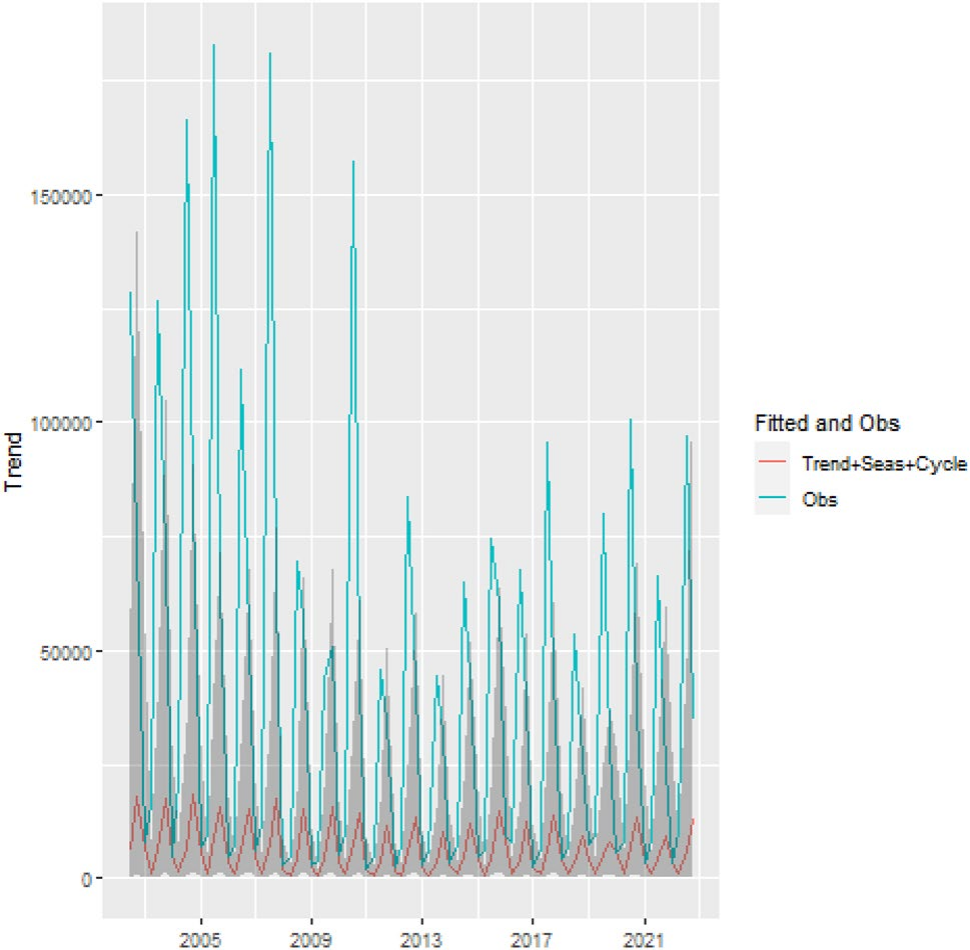
Predicted fires given by the sum of trend, seasonality and cycle components and observed fires. Shaded areas in the graph represent the 95% Bayesian credibility interval.

In order to show the model’s ability to fit the fire occurrence, we plotted the estimated log intensity function and the observed fire occurrence (black dots; see Fig. 5), which shows that the estimated log intensity function explains the spatio-temporal variation observed in the fire count in the Legal Amazon, suggesting that the model has a good fit. Additionally, to show the importance of the trend, seasonal and cycle components in the analysis of fire occurrence in the Legal Amazon, we plotted the observed total fire count and the predicted value of fire count in each year given by the sum of the estimated trend, seasonal, cycle and intercept components (see Fig. 6)

### Amazon biome

The region known as Legal Amazon in Brazil comprises nine Brazilian states, containing three different biomes: Amazon, Cerrado and Pantanal. These biomes differ from each other not only in vegetation and fauna, but in the way they provide ecosystem services. As a complementary analysis, we did the same previous analysis, but now considering the Amazon biome, in order to uncover possible changes in the patterns only in this biome. The results (see Table 2) obtained with the analysis of the Amazon biome differ from the previous one mostly in terms of land cover. While we considered the three biomes in the analysis, the relationship between fire occurrence and wetland, grassland, pasture and soybean were positive. On the other hand, when we consider only the Amazon biome, these relationships become negative.

In the case of wetlands and grasslands, this result can be attributed to the fact that the Pantanal biome is the major wetland ecosystem of the world, characterized by well-defined dry and wet seasons. Also, the Pantanal and Cerrado are biomes in which fire-dependent ecosystems (savanna and grassland) predominates, i.e., in these type of formation fires are typically mild and frequent, often occurring in the transitional months between seasons, mostly during dry seasons, and providing benefits to the fauna and flora^39,40^. Differently from the Pantanal and Cerrado, the Amazon biome is covered predominantly by dense forest formation, which is considered fire-sensitive. As a consequence, in the absence of Pantanal and Cerrado biomes in the analysis, when we consider only the Amazon biome, due to its features, the relationship between the intensity of fire activity and cerrado, savanna, and wetland become negative.

Regarding the pasture and soybean fields, according to^41^, in the Cerrado and Pantanal, the climate is the major determinant of fire activity, while human action is the main driving factor in the Amazon biome. In this case, the incidence of accidental fires in pastures and agricultural areas caused by climate variables is higher in the Pantanal and Cerrado than in the Amazon biome. As a consequence, when we analyze only the Amazon biome, the relationship between the intensity of fire activity and pasture and soybean fields becomes negative.

**Table 2:** Estimated parameters for the Amazon biome.

	Mean	SD	0.025quant	0.5quant	0.975quant	Mode
Fixed effects						
Distance highways	− 0.011	0.001	− 0.014	− 0.011	− 0.009	− 0.011
Rainfall	0.003	0.001	0.002	0.003	0.004	0.003
Köppen 1 (Cwa)	− 0.088	0.431	− 0.932	− 0.088	0.756	− 0.088
Köppen 2 (Am)	0.325	0.065	0.197	0.325	0.454	0.325
Köppen 3 (Af)	0.357	0.087	0.186	0.357	0.529	0.357
Köppen 4 (Cfa)	0.534	0.209	0.123	0.534	0.945	0.534
Köppen 10 (As)	− 0.391	0.158	− 0.701	− 0.391	− 0.082	− 0.391
Köppen 12 (Aw)	0.238	0.136	− 0.028	0.238	0.503	0.238
Forest formation	− 0.094	0.158	− 0.404	− 0.094	0.215	− 0.094
Savanna formation	0.001	0.169	− 0.331	0.001	0.333	0.001
Mangrove	0.298	0.252	− 0.196	0.298	0.792	0.298
Forest plantation	0.530	0.561	− 0.569	0.530	1.629	0.530
Wetland	− 0.418	0.195	− 0.799	− 0.418	− 0.037	− 0.418
Grassland	− 0.105	0.165	− 0.430	− 0.105	0.219	− 0.105
Pasture	− 0.071	0.159	− 0.383	− 0.071	0.241	− 0.071
Beach, dune and sand spot	0.064	0.234	− 0.394	0.064	0.522	0.064
Urban area	0.532	0.571	− 0.587	0.532	1.651	0.532
Salt flat	− 0.146	0.358	− 0.848	− 0.146	0.556	− 0.146
River, lake and ocean	0.101	0.156	− 0.204	0.101	0.407	0.101
Soybean	− 0.053	0.181	− 0.408	− 0.053	0.302	− 0.053
Other temporary crops	0.042	0.189	− 0.328	0.042	0.413	0.042
Random effects						
Precision for trend	5.622	0.453	4.817	5.591	6.604	5.506
Precision for seasonality	0.421	0.034	0.361	0.418	0.493	0.411
PACF4 for seasonality	0.769	0.016	0.739	0.768	0.801	0.765
Precision for cycle	4.033	0.408	3.270	4.020	4.875	4.007
PACF1 for cycle	0.149	0.048	0.055	0.148	0.246	0.144
PACF2 for cycle	− 0.379	0.057	− 0.478	− 0.383	− 0.255	− 0.398
Log $\tau$	− 2.157	0.014	− 2.182	− 2.157	− 2.129	− 2.158
Log $\kappa$	− 0.125	0.012	− 0.148	− 0.126	− 0.099	− 0.129
Group $\Phi$	0.873	0.004	0.863	0.873	0.881	0.874

**Figure 7:**
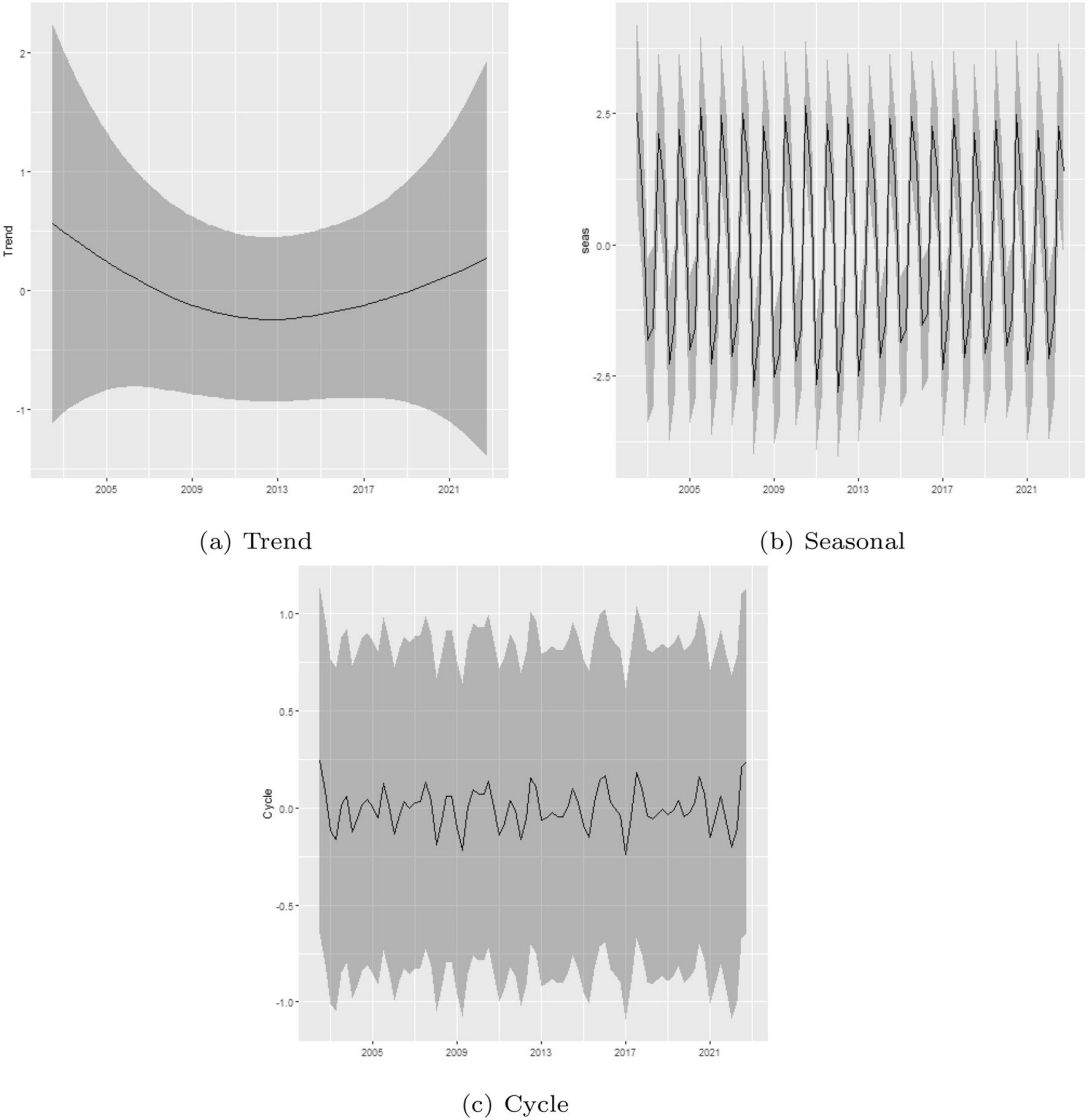
Trend, seasonal and cycle decomposition of fire occurrences in the Amazon biome.

Figure 7 shows the posterior mean and 95% Bayesian credibility interval for the estimated trend, seasonal and cycle components considering the data for the Amazon biome. As the previous result, the trend component also exhibits a marked decrease between the beginning of the sample and 2012, followed by an increase that extends to the end of the sample. Similarly, the cycle and seasonal components are also quite stable, and the model does not indicate relevant changes in those components.

The spatial heterogeneity of the fire occurrence in the Amazon biome (see Figure 1 in Supplementary Material) is also very similar to the previous analyze, as well as the estimated log intensity function and the observed fire occurrence (black dots; see Figure 2 in Supplementary Material), and the observed total fire count and the predicted value of fire count in each year given by the sum of the estimated trend, seasonal, cycle and intercept components (see Figure 3 in Supplementary Material).

### Monthly data

**Figure 8:**
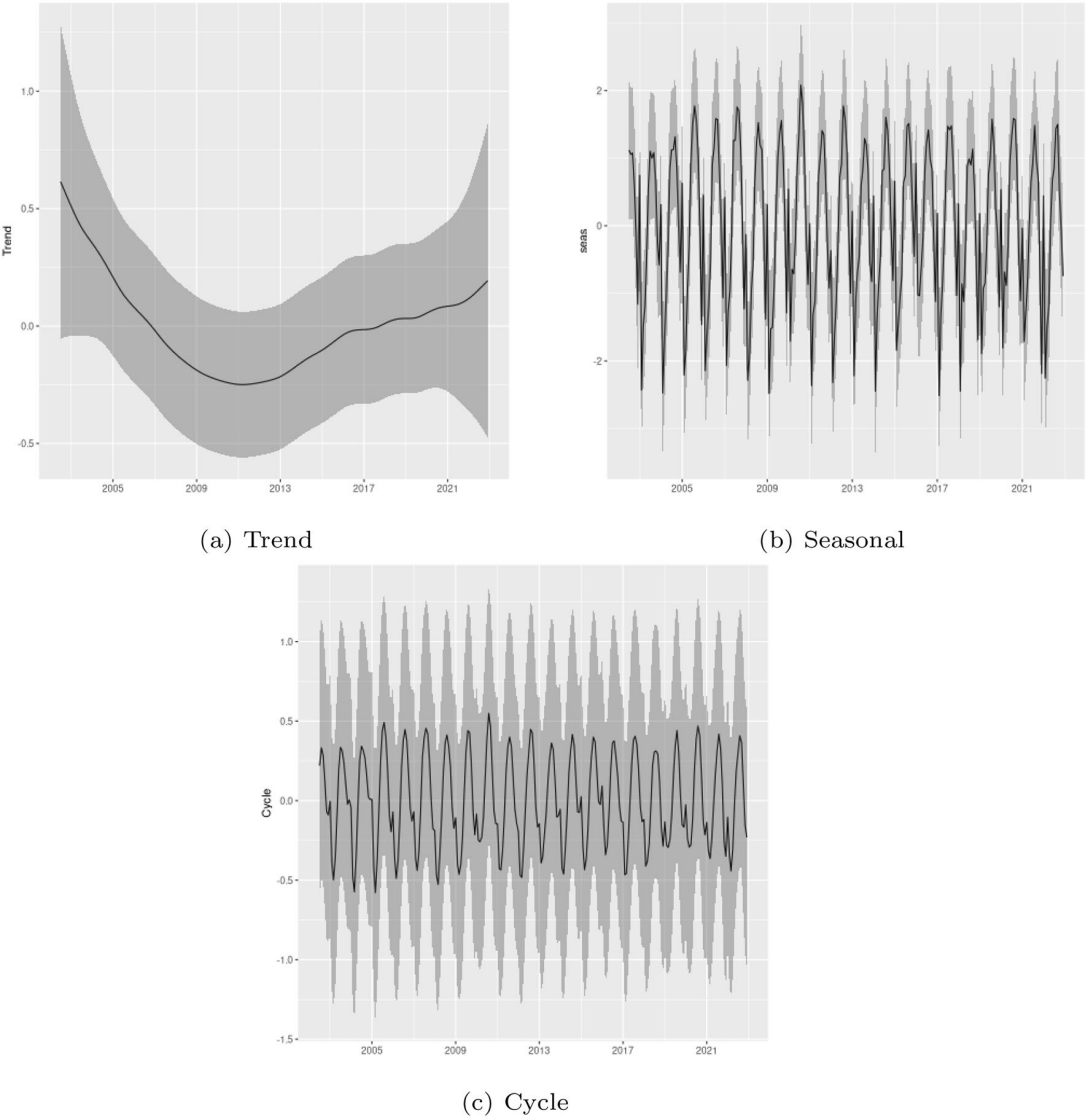
Trend, seasonal and cycle decomposition of fire occurrences in the Legal Amazon-monthly data.

In order to consolidate our results, we also provide a monthly analysis of the changes in the patterns of fire intensity in the Legal Amazon, and the results are presented in Table 2 in Supplementary Material. With respect to the estimated trend, seasonality, and cycle components, showed in Fig. 8, the monthly analysis revealed the same patterns observed in the previous analysis, however, as expected, with less uncertainty. Lastly, considering the observed total fire count and the predicted value of fire count in each year given by the sum of the estimated trend, seasonal, cycle and intercept components (see Figure 4 in Supplementary Material), it also shows the importance of the trend, seasonal and cycle components in the analysis of fire occurrence in the Legal Amazon.

## Discussion

According to our findings, the intensity of fire occurrence in the Legal Amazon, encompassing the Amazon, Cerrado, and Pantanal biomes, as well as specifically in the Amazon biome, exhibits temporal and spatial variability. These results align with the findings presented by Libonati et al. (2021)^42^, who conducted an analysis of the interconnections between deforestation, fire, and droughts in the Brazilian Amazon.

Our examination of the trend component indicates a decline in fire activity from 2002 to 2012. This decrease can primarily be attributed to the implementation of governance measures and market mechanisms. Notably, the policies implemented to mitigate deforestation underwent significant revisions during the 2000s, introducing innovative procedures for monitoring, environmental control, and territorial management. For instance, the Action Plan for the Prevention and Control of Deforestation in the Legal Amazon (PPCDAm), launched its initial and highly successful phase in 2004. Furthermore, novel policy measures were introduced in 2008, specifically targeting municipalities with high deforestation rates and implementing restrictions on rural credit^43^.

The effectiveness of these policies and the impact of market mechanisms on deforestation in the Legal Amazon have been extensively discussed in the literature. Numerous studies have demonstrated that conservation policies, coupled with decreases in agricultural prices and the availability of rural credit, have effectively curbed deforestation^43–48^.

Furthermore, our analysis revealed a notable trend starting from 2013, indicating a continuous increase in the long-term component. This trend seemingly corresponds to the expanding opportunities in international markets for Brazilian beef and soy, which exerted greater pressure on forested areas during this period. Another contributing factor was the replacement of Brazil’s Forest Code (Law 4771/1965) by Law 12651/2012. This legislative change resulted in reduced restrictions and pardoned areas that had been illegally cleared prior to 2008, leading to significant environmental and social challenges^49^.

By incorporating covariates into our statistical model, we were able to examine the associations between the intensity of fire occurrences, human activities, and climate dynamics. Fire occurrence typically depends on four key factors: the presence of sufficient biomass, availability of burnable biomass, conducive ambient conditions for fire spread, and ignition sources^50^. These conditions are influenced by meteorological patterns and their interaction with vegetation types. Our results indicate that climate patterns and human actions play pivotal roles in driving the observed trend of fire occurrences.

In support of the notion that fire intensity is linked to climate variability, our analysis provides evidence of a positive dependence between temperature and fire activity, as well as a negative relationship between rainfall and fire events. Furthermore, human impacts can amplify the influence of biophysical drivers through actions like altering land use, igniting fires, and suppressing fire occurrences^51^.

Within this context, our results offer evidence that pasturelands, areas with mixed land uses, soybean fields, and other temporary crops exhibit a positive correlation with fire intensity. Conversely, non-vegetated areas demonstrate a negative relationship with fire occurrence. It is important to acknowledge that for thousands of years, humans have actively manipulated fire regimes. They have suppressed wildfires as a means to safeguard lives and property, resulting in landscapes that inhibit the widespread propagation of fires. Consequently, these anthropogenic influences lead to fire regimes that differ in terms of frequency, seasonality and intensity, from the natural fire patterns in the absence of human intervention^52,53^.

From a spatial perspective, it becomes apparent that the spatial random effects effectively capture the variability within the Legal Amazon region. This variability is particularly pronounced in regions classified as wet tropical (Am), which experience a dry season between August and November (third and fourth quarters), as well as in areas categorized as tropical with a dry season (Aw). Conversely, in the western Amazon, characterized by a predominantly tropical climate without a dry season (Af), the level of variability is relatively lower.

## Conclusion

The Amazon biome plays a crucial role in the climate system, exerting both regional and global influences. Fire occurrences, resulting from natural and human activities, are significant disturbances in the Legal Amazon, causing notable impacts. Extensive literature highlights changes in the patterns of fire occurrence in the Amazon region, attributed to various factors such as dry conditions, deforestation, agricultural expansion, climate change, and climatic anomalies like El Niño events.

The objective of this study was to examine potential changes in fire occurrence patterns in the Legal Amazon, utilizing a spatio-temporal point process framework. To enable inference procedures, we proposed a structural decomposition approach for analyzing spatio-temporal point pattern data. Specifically, we introduced a dynamic representation of a Log Gaussian Cox process, which models the intensity function through the decomposition of components including trends, seasonality, cycles, covariates, and spatial effects. This formulation effectively captured permanent changes, as well as seasonal and cyclic effects. Moreover, the utilization of a Bayesian hierarchical structure facilitated computationally efficient inference within the integrated nested Laplace approximation framework.

We present the results obtained from analyzing fire occurrence data in the Legal Amazon, specifically reported by MODIS, spanning from July 2002 to December 2022. To account for key fixed effects related to climatic conditions and agricultural practices, we incorporated explanatory variables. Our findings reveal a notable decline in the estimated trend component of fire occurrence from 2002 to 2012, followed by a subsequent increase that persisted until the end of the dataset. These patterns could be linked to governance actions and market mechanisms. Additionally, our model effectively captured the variability within the Legal Amazon, particularly in regions classified as wet tropical (Am), characterized by a dry season occurring between August and November (third and fourth quarters), as well as tropical regions with a dry season (Aw). Conversely, in the western Amazon, characterized by a predominantly tropical climate without a dry season (Af), variability was comparatively low.

### Supplementary Information

Supplementary Information.

## Data Availability

All data is from public sources, as detailed in the manuscript.

## Code availability

Custom code developed by the authors, using r-inla (www.r-inla.org) library.
