# Supplementary material for: A spatio-temporal analysis of fire occurrence patterns in the Brazilian Amazon
Source: Sci Rep. 2023 Aug 5;13:12727. doi: 10.1038/s41598-023-39875-z (PMC10404243; doi:10.1038/s41598-023-39875-z)
Supplement: Supplementary file 1 — Supplementary Information. [file 41598_2023_39875_MOESM1_ESM.pdf]

Supplementary Material - Mapping Changes in Fire Occurrence in the Brazilian Amazon: A Spatio-Temporal Analysis

Climate classification

Table 1: Köppen Climate Classification

**Characters**	**Description**
Cwa	(C) Humid subtropical (w) With dry winter (a) and hot summer.
Am	(A) Tropical (m) monsoon.
Af	(A) Tropical (f) without dry season.
Cfa	(C) Humid subtropical (f) Oceanic climate, without dry season (a) and hot summer.
Cwb	(C) Humid subtropical (w) With dry winter (b) and temperate summer.
Csb	(C) Humid subtropical (s) With dry summer (b) and temperate summer.
Csa	(C) Humid subtropical (s) With dry summer (a) and hot summer.
Cfb	(C) Humid subtropical (f) Oceanic climate, without dry season (b) and temperate summer.
BSh	(B) Dry (S) Semi-arid (h) low latitude and altitude.
As	(A) Tropical (s) with dry summer.
Cwc	(C) Humid subtropical (w) With dry winter and (c) short and cool summer.
Aw	(A) Tropical (w) with dry winter.

Amazon biome - Additional results

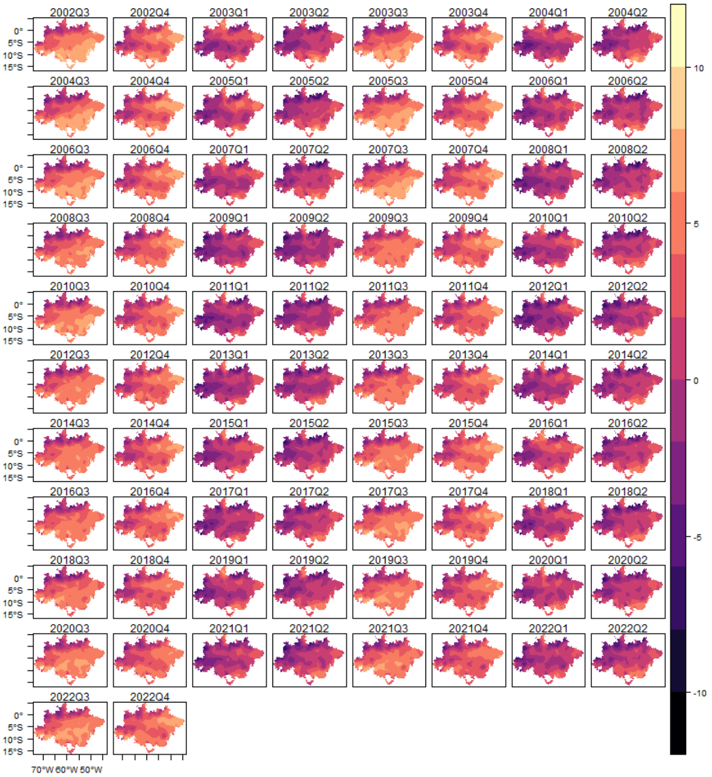

Fig. 1: Spatial Random Effects - Amazon biome

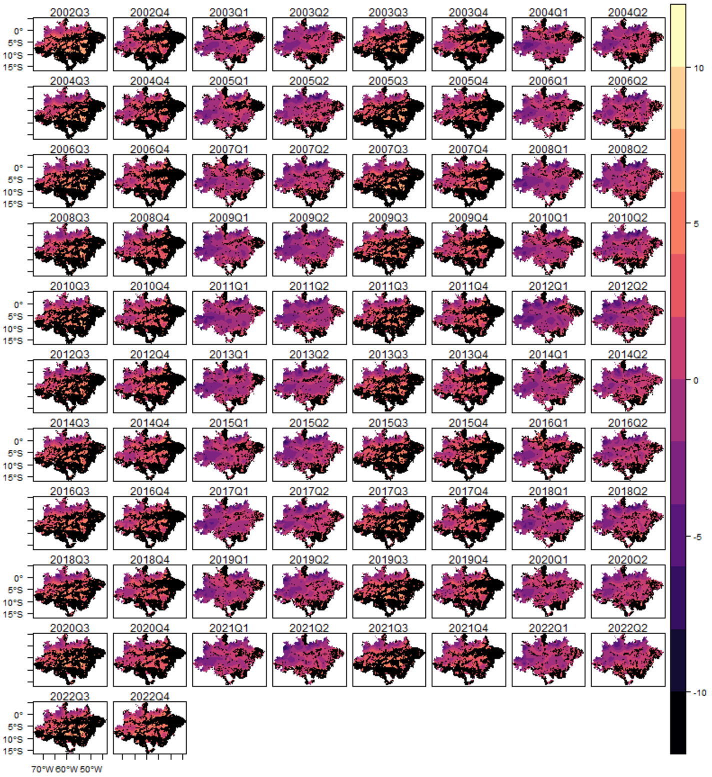

Fig. 2: Estimated log-intensity function and observed fire occurrence - Amazon biome

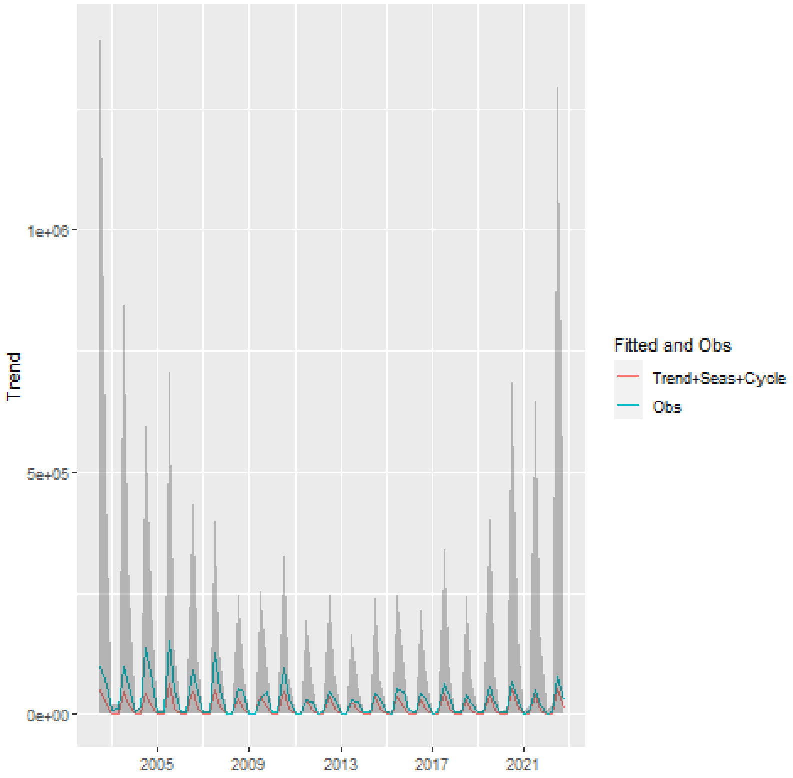

Fig. 3: Predicted fires given by the sum of trend, seasonality and cycle components and observed fires for the Amazon biome. Shaded areas in the graph represent the 95% Bayesian credibility interval.

Monthly data - Additional results

Table 2: Estimated Parameters for the Legal Amazon - Monthly data

	Mean	SD	0.025quant	0.5quant	0.975quant	Mode
Fixed effects						
Distance Highways	-0.013	0.001	-0.015	-0.013	-0.012	-0.013
Temperature	0.086	0.005	0.076	0.086	0.096	0.086
Rainfall	-0.014	0.001	-0.016	-0.014	-0.012	-0.014
Köppen 1 (Cwa)	-0.061	0.181	-0.415	-0.061	0.294	-0.061
Köppen 2 (Am)	0.306	0.044	0.220	0.306	0.391	0.306
Köppen 3 (Af)	0.030	0.065	-0.098	0.030	0.158	0.030
Köppen 4 (Cfa)	0.237	0.206	-0.167	0.237	0.642	0.237
Köppen 10 (As)	0.312	0.113	0.091	0.312	0.533	0.312
Köppen 12 (Aw)	0.736	0.090	0.559	0.736	0.913	0.736
Forest Formation	0.037	0.041	-0.043	0.037	0.117	0.037
Savanna Formation	0.140	0.062	0.019	0.140	0.261	0.140
Mangrove	0.085	0.111	-0.133	0.085	0.303	0.085
Wetland	0.243	0.090	0.065	0.243	0.420	0.243
Grassland	0.264	0.058	0.150	0.264	0.378	0.264
Pasture	0.211	0.059	0.097	0.211	0.326	0.211
Mosaic of Uses	0.302	0.108	0.091	0.302	0.513	0.302
Beach, Dune and Sand Spot	0.651	0.184	0.291	0.651	1.011	0.651
Other non Vegetated Areas	-0.617	0.157	-0.924	-0.617	-0.310	-0.617
River, Lake and Ocean	0.235	0.049	0.140	0.235	0.330	0.235
Soybean	0.372	0.135	0.107	0.372	0.638	0.372
Other Temporary Crops	0.251	0.144	-0.031	0.251	0.533	0.251
Random Effects						
Precision for trend	5.402	0.115	5.144	5.411	5.588	5.468
Precision for seasonality	0.908	0.013	0.883	0.908	0.936	0.906
PACF4 for seasonality	0.067	0.009	0.048	0.068	0.083	0.070
Precision for cycle	4.862	0.092	4.689	4.858	5.057	4.844
PACF1 for cycle	0.341	0.009	0.321	0.342	0.356	0.345
PACF2 for cycle	-0.349	0.008	-0.366	-0.349	-0.334	-0.347
Log τ	-2.250	0.006	-2.265	-2.249	-2.240	-2.245
Log κ	0.286	0.004	0.280	0.285	0.294	0.284
Group Φ	0.853	0.002	0.850	0.852	0.856	0.851

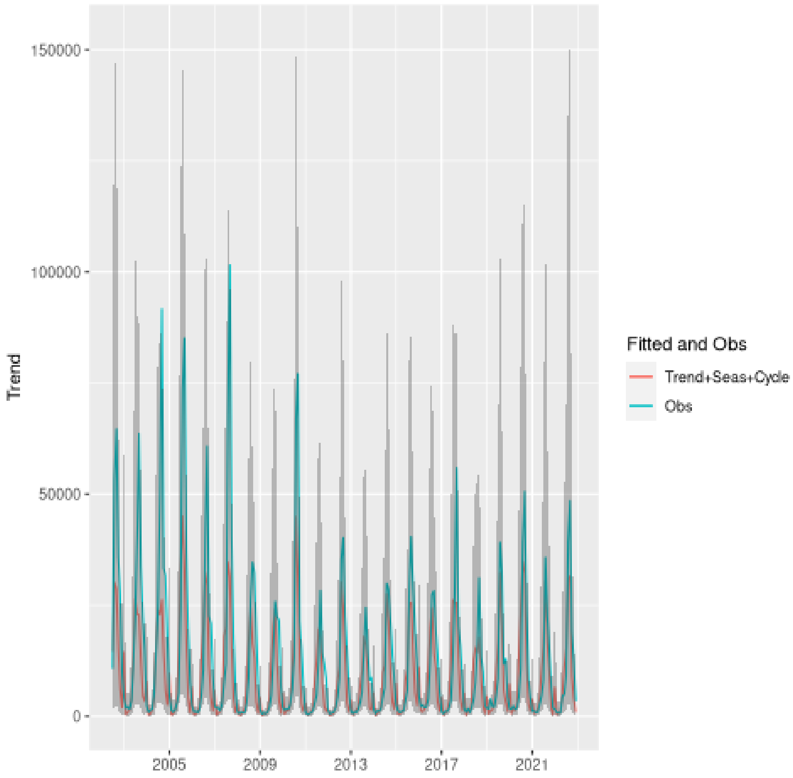

Fig. 4: Predicted fires given by the sum of trend, seasonality and cycle components and observed fires using monthly aggregation. Shaded areas in the graph represent the 95% Bayesian credibility interval.
